# Infectious diseases prevention and control using an integrated health big data system in China

**DOI:** 10.1186/s12879-022-07316-3

**Published:** 2022-04-06

**Authors:** Xudong Zhou, Edmund Wei Jian Lee, Xiaomin Wang, Leesa Lin, Ziming Xuan, Dan Wu, Hongbo Lin, Peng Shen

**Affiliations:** 1 The Second Affiliated Hospital, Zhejiang University School of Medicine, Hangzhou, 310058 China; 2 Institute of Social & Family Medicine, Zhejiang University School of Medicine, 866 Yuhangtang Road, Hangzhou, 310058 China; 3 Wee Kim Wee School of Communication and Information, Nanyang Technological University, 31 Nanyang Link, WKWSCI Building, Singapore, 637718 Singapore; 4 Laboratory of Data Discovery for Health (D24H), Hong Kong Science Park, Hong Kong Special Administrative Region, China; 5 Department of Infectious Disease Epidemiology, London School of Hygiene & Tropical Medicine, London, WC1E 7HT UK; 6 Department of Community Health Sciences, Boston University School of Public Health, 801 Massachusetts Ave, Boston, MA 02118 USA; 7 Department of Clinical Research, London School of Hygiene & Tropical Medicine, London, WC1E 7HT UK; 8 Yinzhou Center for Disease Prevention and Control, 1221 Xueshi Road, Ningbo, 315100 Zhejiang China

**Keywords:** Electronic health records, Big data analytics, Infectious disease, Dengue, Pulmonary tuberculosis, Immunization

## Abstract

**Background:**

The Yinzhou Center for Disease Prevention and Control (CDC) in China implemented an integrated health big data platform (IHBDP) that pooled health data from healthcare providers to combat the spread of infectious diseases, such as dengue fever and pulmonary tuberculosis (TB), and to identify gaps in vaccination uptake among migrant children.

**Methods:**

IHBDP is composed of medical data from clinics, electronic health records, residents’ annual medical checkup and immunization records, as well as administrative data, such as student registries. We programmed IHBDP to automatically scan for and detect dengue and TB carriers, as well as identify migrant children with incomplete immunization according to a comprehensive set of screening criteria developed by public health and medical experts. We compared the effectiveness of the big data screening with existing traditional screening methods.

**Results:**

IHBDP successfully identified six cases of dengue out of a pool of 3972 suspected cases, whereas the traditional method only identified four cases (which were also detected by IHBDP). For TB, IHBDP identified 288 suspected cases from a total of 43,521 university students, in which three cases were eventually confirmed to be TB carriers through subsequent follow up CT or T-SPOT.TB tests. As for immunization screenings, IHBDP identified 240 migrant children with incomplete immunization, but the traditional door-to-door screening method only identified 20 ones.

**Conclusions:**

Our study has demonstrated the effectiveness of using IHBDP to detect both acute and chronic infectious disease patients and identify children with incomplete immunization as compared to traditional screening methods.

## Background

The advent of big data platforms and advancements in machine learning algorithms have allowed researchers to distil insights from large amounts of information, which has the potential to create increasingly effective infectious diseases surveillance and control methods [1]. The term *big data* refers to complex datasets that are too voluminous and sophisticated to be handled by traditional analytics [2, 3] and public health agencies are increasingly reliant on big data to improve infectious disease screening. There is a wide variety of data structures that are relevant to public health concerns, including electronic health records (EHRs), clinical databases at the state and local level, social media, and reports from public health agencies such as the Centers for Disease Control and Prevention (CDC) [4]. The ease of storing, manipulating, and analyzing these wide ranging data types at scale has potential to empower health organization and public health officials to preempt the spread of infectious diseases and respond to and manage outbreaks in a timely manner [5].

While researchers have recognized the promises of big data in enhancing infectious disease monitoring and control, the efficacy of big data in preparing public health officials at both the state and national levels to respond to infectious diseases outbreak is largely contingent on the existence of successful partnerships between different stakeholders, such as government health agencies, departments in infectious disease control, and private and public hospitals and clinics. A concerted effort in storing, transferring, manipulating, and analyzing data efficiently for infectious disease control requires the support of computational and organizational infrastructures and the streamlining of processes between different stakeholders.

The overall objective of this study is to demonstrate how building an integrated, big data health infrastructure and platform at the district-level can significantly improve public health officials’ ability to leverage insights from big data for infectious disease prevention and control. To achieve this objective, we compared the effectiveness of a new tool with an integrated big data platform located in Yinzhou, a major district in the city of Ningbo in Zhejiang province in China [6], with a traditional infectious disease surveillance method—which relies on reporting from laboratories and healthcare facilities (e.g., doctors’ diagnosis after face-to-face consultation)—in case identification for infectious disease control and prevention. For this study, we focused on three types of infectious diseases categories: (a) acute infectious diseases (i.e., dengue fever), (b) chronic infectious diseases (i.e., pulmonary tuberculosis (TB)), and (c) gaps in vaccination uptake among migrant children.

### Challenges of traditional infectious disease surveillance methodologies

Prior to the introduction of big data, public health officials traditionally engaged in infectious disease monitoring and control in three ways. The first was reliance on doctors to report when they came across suspected cases. These reports would then be compiled and sent to a centralized infectious disease agency (e.g., CDC) for coordinating control and prevention measures. Secondly, public health officials relied on forecasting models drawn from sources such as meteorological and vector surveillance data [7, 8], or in recent years social media sources [4]. Thirdly, public health officials would rely on immunization records to examine vaccination uptake. This would enable government and public health agencies to target specific populations that remain unvaccinated and who are therefore most at-risk from outbreaks.

However, these traditional approaches have several limitations. First, there is a high probability of human error in diagnosing infectious disease, even if the diagnosis was done by highly qualified medical doctors. For instance, research has shown that a substantial portion of TB patients had been diagnosed with a non-TB respiratory-related diagnosis in hospitals [9]. In low resource healthcare settings, physicians may sometimes misdiagnose dengue fever patients and treat them as having a common upper respiratory tract infection. The inaccuracy of traditional diagnosis methods would be magnified if hospitals and clinics are overwhelmed with a sudden surge in cases.

Second, while forecast modelling using a variety of data sources can potentially aid public health organizations to preempt the spread of infectious diseases, these models have several limitations that inhibit their effectiveness. There are problems in modelling associated with data deluge and hubris, resulting in over or underestimation of statistical models [4]. On the other hand, in areas that are rural and do not have Internet broadband access, there may be problems associated with a lack of quality data [10], where data from underserved communities are not represented, severely compromising the quality of forecasting models.

Third, while identifying vaccination gaps using local immunization records is a powerful step in prevention, the efficacy of this method is diluted if there is an influx of migrants, who might be carriers of infectious diseases if they are not vaccinated. In the context of Yinzhou, immunization records do not capture the vaccination rates of migrant children who are not listed as children within a local urban *hukou*—a system of household registration—in China, thus making it hard to track infectious diseases that originate from other geographical regions. As such, primary care providers in local health facilities are responsible for screening migrant children by going door-to-door to identify those who might not have completed all basic immunizations, resulting in medical facilities taking on huge financial and manpower costs.

### Addressing the challenges—implementation of infectious disease big data platform in Yinzhou

**Fig. 1:**
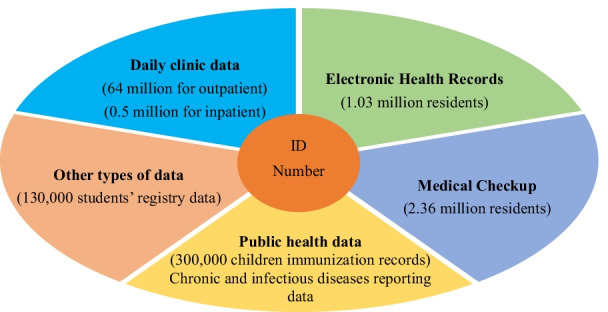
Types of data in Yinzhou’s big data platform

To circumvent the weaknesses in traditional infectious disease prevention and control methods, Yinzhou recently implemented a synchronized district-wide health big data platform to coordinate data collection from local healthcare providers, such as clinics and hospitals, across both the private and public sectors. The big data platform was developed by Wonders Information Co Ltd—commissioned by the CDC—for the purpose of coordinating data sharing among a network of clinics and hospitals for the purpose of public health monitoring by the CDC. The big data system consolidates different data sources such as (a) daily clinic data produced by local health providers, (b) EHRs, (c) annual medical checkup data, (d) public health related data such as immunization records, chronic and infectious diseases reporting data, and mortality surveillance data, and (e) other types of data such as students’ registry data (see Fig. 1). Individuals’ data were linked and connected using their unique ID number and new data entries (e.g., visits to medical facilities) are uploaded onto the big data system daily. The system conducts daily scans for potential cases of infectious diseases among the population, and notifies the relevant public health agencies and authorities for further action.

For this study, we illustrate the accuracy and performance of the integrated big data system in identifying (a) dengue cases, (b) pulmonary TB among university students, and (c) migrant children without complete vaccination records, when compared to traditional surveillance methodologies. While the integrated big data system contains information on all infectious diseases, we specifically chose to focus only on dengue and pulmonary TB as they are the top public health priorities within Zhejiang Province. Located on the eastern coast of China, Zhejiang Province has humid subtropical climate, with heavy rainfall through spring and summer, with an abundance of aedes albopictus [11]. Moreover, the high density of human population, as well as the metropolitan nature of the city with large influx of travelers, the province is susceptible to individuals who import dengue from dengue-endemic regions, which have been the cause of a few dengue outbreaks in recent years [12]. Also, in China’s context, pulmonary TB is a cause for concern as outbreaks often occur in education settings where there are crowded dormitories or close proximity and contact between students in classrooms—in 2018 there 48,289 students were reported to have pulmonary TB, which was an incidence of 17.97/100,000 [13].

## Methods

**Fig. 2:**
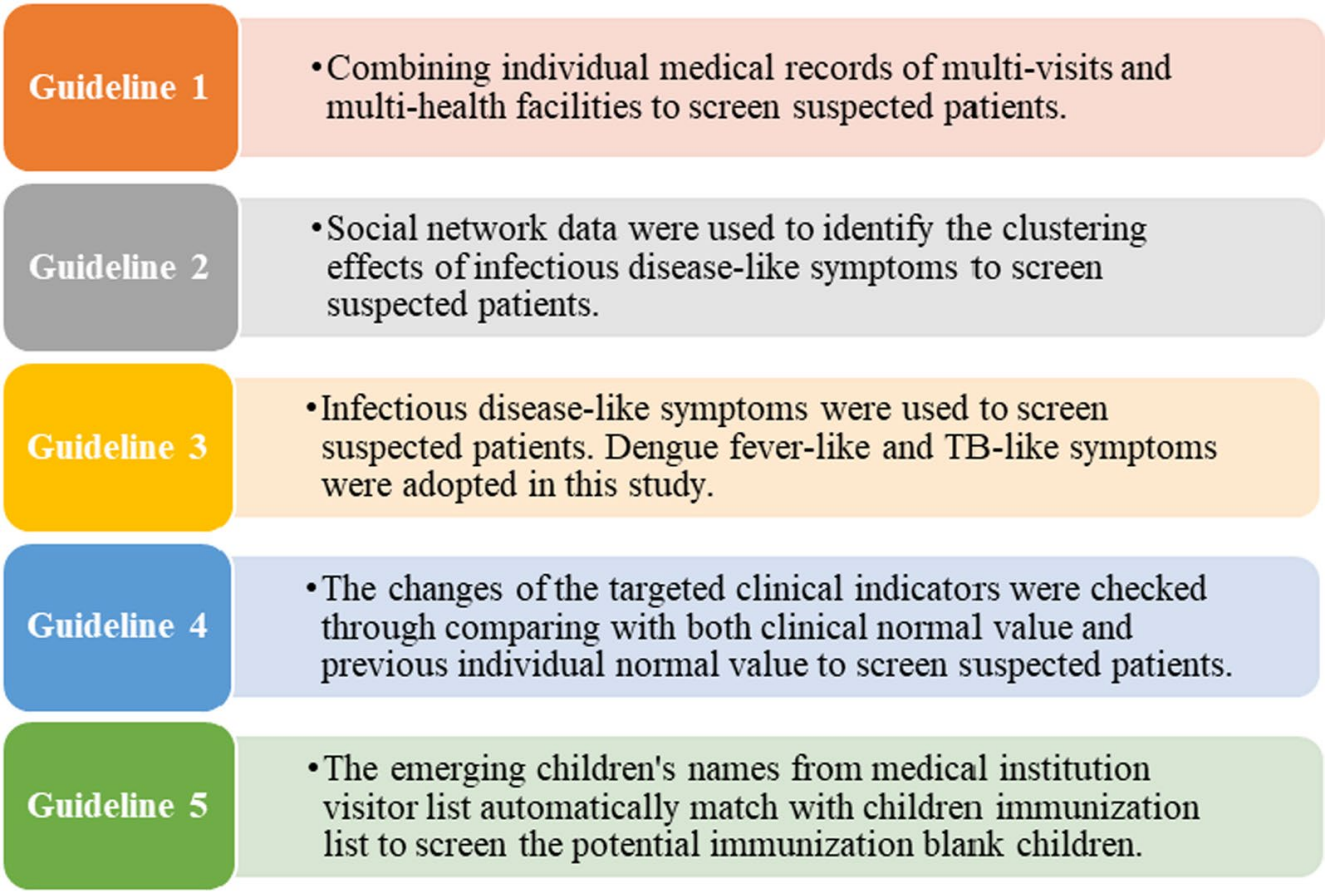
Infectious disease surveillance framework of Yinzhou big data platform

The big data system in Yinzhou was established in 2016 as part of the district’s effort to accelerate and strengthen infectious disease control by pooling together a diverse set of medical and clinical data that health organizations can access for decision-making in a timely manner. To effectively screen for infectious diseases, we developed an overarching infectious disease screening framework with five key guidelines (see Fig. 2) that guide the detection of infectious diseases. These guidelines were derived from best practices and findings from existing research on the use of various forms of big data systems in complementing traditional infectious disease surveillance methods [14–16]. For the purpose of this study, we programmed specific clinical criteria (see Table 1) after consultations with public health and medical experts involved in the big data system and screened for (a) dengue fever among the outpatients, (b) TB among university students, and (c) migrant children with insufficient vaccination coverage. To screen for TB among individuals, we extracted any key unstructured texts in medical imaging diagnosis abstracts for patients with diagnosis of acute respiratory infection as well as cold and pneumonia with the following keywords: shadow; pleural thickening; pleural effusion; lung infection; infective lesion; pulmonary nodules. If the patients were diagnosed with the above symptoms for more than 2 times across 14 days in a month, or more than 5 times in a month, they would be identified as a potential suspect patient. When a student is listed as a suspect case based on the above criteria, we also applied the same screening criteria on students’ social networks in their classes and dormitories to identify potential spread of TB.

**Table 1:** Screening criteria of infectious diseases

Infectious diseases	Guidelines	Screening criteria	Interventions
TB	G1 G2 G3	**A:** Any following diagnoses: acute upper respiratory infection (J00–J06); cold and pneumonia due to certain identified influenza viruses (J09–J18); Acute lower respiratory infection (J20–J22); Other upper respiratory infection (J30–J39) **B:** Any following key unstructured texts in medical imaging diagnosis abstracts: shadow; pleural thickening; pleural effusion; lung infection; infective lesion; pulmonary nodules **Individual patient (in the past month)** **C1:** Diagnoses with A ≥ 2 times; the interval ≥ 14 days **C2:** Diagnoses with A ≥ 5 times **C3:** Diagnoses with A and B **Multi patients (in the past month)** **D1:** The number of roommates with diagnoses A ≥ 2 **D2:** The number of classmates with diagnoses A ≥ 5 **D3:** The number of classmates with diagnoses B ≥ 2 **D4:** The number of students in one school or one grade with diagnoses B ≥ 5 **E:** If a TB patient is confirmed, the students with diagnoses A or B in the same dormitory or class in the past and next 3 months will be included in suspected patients	**Individual patient screening** The patient who satisfies with **C1** or **C2** or **C3** will be screened as a suspected patient **Multi patients screening** (1) The patients who satisfy with **D1** or **D2** or **D3** or **D4** will be screened as suspected patients (2) The patients who satisfy with **E** will be screened as suspected patients The selected suspected patients will be reviewed using *Lung TB Outpatient Diagnosis and Treatment Guideline* by the CDC officials The reviewed suspected patients will be referred to TB specialized hospital to confirm using CT scanning or T-SPOT.TB test
Dengue fever	G3 G4	**A:** Patients > 15 years old **B1:** White blood cell count (WBC) < 4.5 * 10^9^/L **B2:** WBC reduced by 10% compared with the most recent medical record (health check record first, or medical records with normal WBC < 9.5 * 10^9^/L) **C1:** Platelet count (PLT) < 125 * 10^9^/L **C2:** PLT reduced by 10% compared with the most recent medical record (health check record first, or medical records with normal PLT < 350 * 10^9^/L) **D:** In the past 5 days with any following diagnoses: Fever (R50.800; R50.900; A92.800; A92.900; A94.X00; A94.X01); Infectious fever (B99.X01); Viral Infection (B34.800); Upper Respiratory Tract Inflection (J06.90); Acute pharyngitis (J02.80; J02.900); Cold (BNW01); Erythra (R21.X00; B09.X01); Thrombocytopenia (D69.400; D69.403; D69.500; D69.501; D69.600) All the screening criteria were validated using the confirmed dengue fever cases from both Yinzhou and Ningbo from 2014 to 2018 to improve its accuracy and sensitivity	The big data platform ran all the clinical records from health facilities in Yinzhou in the end of a day. The patients who satisfy **A**, **B1** or **B2** or **C1** or **C2**, and **D** will be selected as a suspected dengue fever patient The big data platform automatically returned the suspected patients name list to the original hospitals in the early next day The public health officials of hospital will work with the clinical doctors to confirm the suspected patients including calling the patients to have travel history and other information and re-checking the cases
Migrant children with incomplete immunization	G5	Match the name list of children under 15 years old who visiting medical institutions in Yinzhou with the name list of children who have been covered by local immunization program. Because some younger children did not have an ID number or even a name, we conducted matches as follow: **A:** the ID number of the children **B:** children’s name and birthday **C:** children’s family name, birthday, and township of residence **D:** parents’ names and children’s birthday	If the emerging children can’t match with any cases in the dataset of Yinzhou Immunization Program using **A** and **B** and **C** and **D**, the children will be screened as a potential case with incomplete immunization. The local immunization staff will call the suspected children’s parents and confirm their children’s immunization status

For dengue fever, we screened for patients above 15 years old, with white blood cell count (WBC) < 4.5 * 10^9^/L, or reduced by 10% compared with the most recent medical record; Platelet count (PLT) < 125 * 10^9^/L; PLT reduced by 10% compared with the most recent medical record; or In the past 5 days with any following diagnoses: fever, infectious fever, viral infection, upper respiratory tract infection, acute pharyngitis, cold, and thrombocytopenia.

For screening of migrant children with incomplete immunization, we first matched the name list of children under 15 years old visiting medical institutions in Yinzhou with the name list of children who have been covered by local immunization program by matching the ID number of children, children’s name and birthday, children’s family name, township of residence, and parents’ names and children’s’ birthday. If the children’s records don’t match with any of the cases in the dataset of Yinzhou Immunization program, the children will be identified as a potential case with incomplete immunization.

We then compared the effectiveness of big data screening done with existing traditional methods of detecting TB, dengue, and the number of migrant children without adequate vaccinations.

### 1. Screening of dengue fever patients

In Yinzhou, most dengue cases are imported by people who travel to other geographic regions with an ongoing outbreak [17, 18], as such dengue outbreaks are often sporadic and unpredictable. The traditional way of tracking dengue fever in Yinzhou was through doctors’ diagnosis during face-to-face consultation with patients. Once a patient was confirmed by a doctor to have contracted dengue, this information would then be reported to the Yinzhou CDC. In contrast, the big data system screened for suspected dengue cases automatically, based on the disease symptoms criteria in Table 1. The integrated big data platform automatically returned the suspected patients’ names to their respective hospitals the next day. Public health officials in these hospitals then worked with doctors to confirm if the patients had dengue. We then compared the number of confirmed cases detected by the big data system and through the traditional method.

### 2. Screening of university students with TB

The traditional method of screening for TB among university students (n = 43,521) in Yinzhou was to rely on manual reporting through the collection of health information from the mandatory medical checkup (Purified Protein Derivative skin test, X-ray scans) for newly enrolled university students, or through diagnosis and reports by doctors during consultations. Once a TB diagnosis in a student was confirmed, CDC officials would engage in contact tracing of close contacts among his/her roommates and classmates [19]. In addition to traditional screenings, we programmed the screening criteria of TB—illustrated in Table 1—into the big data system to automatically scan and identify suspected TB cases. If individual student or multiple students met the screening criteria of TB, they would be reviewed using *Lung TB Outpatient Diagnosis and Treatment Guideline 2012* [20] by the CDC officials. The reviewed patients are then referred to a TB-specialized hospital to confirm their diagnosis using CT scanning and the T-SPOT.TB test. If a student was confirmed to have contracted TB, the big data system would then screen the health information of his or her close contacts, such as classmates living in their dormitories [21].

### 3. Screening of migrant children who require vaccination

The traditional method of identifying migrant children with incomplete vaccinations was through the township vaccination department, where the staff would visit migrant children to perform door-to-door screenings (August 2017 to July 2018). From August 2018 onward, we leveraged our big data platform to screen migrant children for incomplete vaccination records, where data were collected when they visited medical facilities (August 2018 to July 2019) using the criteria and procedures shown in Table 1.

### Data protection and privacy

Due to the sensitive nature of healthcare data in Yinzhou, there are several layers of stringent data protection mechanisms built into Yinzhou’s integrated big data system to ensure continual data protection and privacy. In terms of physical infrastructure, the data were stored in the local mobile Internet Database Connector (IDC) which had high security level certification and protection. Privacy protection has been accorded to an individual to control both access to and use of personal information including de-identification and/or desensitization of personal and private information. To safeguard data utilization security, only pre-approved users are permitted to access the database online using the docking stations where regular security audits are conducted to detect unauthorized access. The ethnical committee at School of Public Health, Zhejiang University approved this study (Reference number ZGL201905-5) and waived the requirement for informed consent. All methods were performed in accordance with the relevant guidelines and regulations.

## Results

### Dengue fever screening

Table 2 shows that a total of six dengue fever patients detected in Yinzhou in 2019 were identified by the big data platform. Four patients were detected by both the big data system and the traditional method, but two were missed by the traditional surveillance method. The integrated big data platform did not miss any dengue cases.

**Table 2:** The dengue fever cases detected by Big Data and the traditional way in 2019

Total suspected cases screened by *Big Data*	Confirmed cases detected by both *Big Data* and the traditional way	Confirmed cases detected by *Big Data*, but missed by the traditional way	Confirmed cases detected by the traditional way but missed by *Big Data*
3972	4	2	0

### TB screening among university students

Table 3 shows that the big data platform screened 288 suspected TB patients among 43,521 university students in Yinzhou. Among them, 30 were confirmed as suspected patients after further inspection by Yinzhou CDC officials, and referred to a TB specialized hospital and three were confirmed by CT scan and T-SPOT.TB test. The traditional screening method missed all three cases identified by the big data platform.

**Table 3:** The TB patients screening among university students by Big Data

Total university students	Suspected patients screened by *Big Data*	Suspected patients after CDC checking	Confirmed patients by CT or T-SPOT.TB tests
43,521	288	30	3

### Screening of migrant children with incomplete immunization

Table 4 shows that a total of 983,000 children visited medical institutions from August 2018 to July 2019. Our big data system flagged 11,900 children as potential cases with incomplete vaccination records (using the criteria in Table 1), and ultimately identified 240 children with incomplete vaccination records. In contrast, the door-to-door survey conducted by the township vaccination department—who checked one to two suspected cases daily—only found 20 children with incomplete vaccinations.

**Table 4:** Migrant children with incomplete immunization screened by the *Big Data* and the traditional way

Models (Year)	Total number of children visiting medical institutions (thousand)	The number of suspected children with incomplete or blank immunization (thousand)	The average number of suspected children checked by each township vaccination dept. per day	The number of children confirmed with incomplete or blank immunization
Big Data (Aug, 2018–Jul, 2019)	983	11.9	1.64	240
Traditional way (Aug, 2017–Jul, 2018)	–	–	–	20

## Discussion

Our study has indicated the potential of having a coordinated health big data system in drawing upon different health sources to identify individuals who contracted dengue fever and TB, thereby preempting their further spread, as well as engaging in preventive infectious disease control by identifying high-risk populations who require vaccinations. We conducted our study in Yinzhou district in Zhejiang province in China, which was suitable as it has successfully implemented a health big data system where the health data of individuals (e.g., daily clinic data produced by local health providers, EHRs, annual medical check-up data, immunization records, chronic and infectious diseases reporting data, mortality surveillance data, students’ registry data) are uploaded into a secured centralized EHR on a daily basis. This allows public health officials to have timely access to the latest data in tracking and mitigating the spread of infectious diseases.

In the big data system, all the screening criteria were set by clinical and public health workgroups and calibrated based on consultations with public health and medical professionals. Disease-like symptoms, such as acute upper and lower respiratory infection, cold and pneumonia, pleural thickening, pleural effusion, lung infection, infective lesion, and pulmonary nodules, were used to screen for TB in our big data system. As for dengue fever, we used symptomatic indicators such as fever, upper respiratory tract infection, acute pharyngitis, cold, erythra, and thrombocytopenia. Leveraging disease-like symptoms within the big data system for the screening of infectious diseases—a form of syndromic surveillance which often involves the usage of clinical data to scan for discernable symptoms to identify potential cases of infectious diseases before official diagnosis—was shown to be effective as early as the 1990s [22]. For instance, research has shown that reliance on pre-diagnostic signs and symptoms allows public health organizations to detect community wide influenza outbreaks [23]. This is because the systematic collection of continuous population health data (e.g., whenever people visit medical facilities) through the big data system ensures that the available data are the most up to date, thus bringing it closer to a “real-time” detection of outbreaks [24]. Moreover, they are arguably more accurate, as they do not rely on individuals’ self-reporting or participation through digital communication technologies (e.g., smartphones, web-search queries) [25], which suffer from problems such as non-representativeness, missing data, and mis- or under-reporting [10].

In addition to relying on individual-level disease symptoms, our screening included social network data of university students in an ethical and secure way. Only when a student was suspected or diagnosed with TB would the big data system scan the symptoms of their close contacts, which could be classmates or roommates in our records, for any medical anomalies. Social networks are crucial for understanding and tracking the spread of public health problems and diseases. This was most notably demonstrated by Christakis and Fowler, who modelled the spread of obesity in a large social network over three decades [26]—due to underlying tie-generative mechanisms in social networks. Some of these tie-generative mechanisms [27] are *assortative mixing* (i.e., people who are like-minded would be in closer proximity) and *triadic closure*—a situation where if node *a* is connected to node *b* and node *b* to node *c*, then node *a* is likely to be connected to node *c*, which explains the spread of diseases. The traditional method will initiate an investigation only when a student with TB was diagnosed, while the big data method can automatically screen for students with TB-like symptoms; the traditional method tracks the close contacts within a small scope, such as classmates or roommates, compared to the large scope captured by the big data method, such as students in the same grade; the traditional method investigates the TB-like symptoms of close contacts currently and in the past few weeks, and may be affected by recall bias, whereas the big data method analyses the 3 month period before and after detection.

Using our big data approach, we successfully identified six cases of patients who contracted dengue fever out of a pool of 3972 suspected cases in 2019, three cases of TB out of 288 suspected cases in a pool of 43,521 university students, and 240 children who were without immunization out of a total of 983,000 children. As such, using an integrated big data health system could significantly improve upon the efficiency, time-related costs, and misdiagnoses that come with reliance on manual contact-tracing alone.

There are several advantages to implementing an integrated big data system for infectious disease prediction and control. Through our three case studies, we have illustrated that reliance on big data is effective, timely, and may potentially result in significant cost savings (i.e., it is relatively expensive to send out public health officials to go door-to-door for screening). In addition to these advantages, because the data are located within a geographical boundary (e.g., Yinzhou), this approach allows public health officials working in that region to take practical action. As such, this is better than relying on other forms of “big data” epidemiology, such as the use of web-search queries and social media posts in predicting infectious diseases. There exist multiple confounders in the use of social media posts and web-search queries as proxies for the spread of infectious diseases. Even if researchers were able to identify and model the spread of diseases using user-generated content on social media platforms, public health researchers would not be able to take advantage of the information strategically and practically for their region.

This study has several limitations. First, we relied on aggregated data from Yinzhou CDC for this study and due to the data limitations, we were not able to conduct formal statistical testing to compare the efficacy of the integrated big data system and traditional infectious disease surveillance method beyond that of descriptive comparison. Second, for the three case studies, we did not rely on all possible data types, such as records from pharmacies, social media or web-search data. While social media and web-search data are meaningful in forecasting [28, 29], we have chosen to exclude them as the purpose of the study is to examine the effectiveness of the big data system in identifying potential cases, while social media and web-search queries are more effective in aggregated forecast. While our case studies were specific to dengue, TB, and immunization records, we are cognizant of the limitations of generalizability and scalability of this method to other infectious diseases, especially in the light of emerging pandemics such as COVID-19. Finally, we are mindful that individuals’ health data are highly sensitive and at-risk of malicious cyber-attacks—however we have put in place substantial cyber security measures to protect this sensitive data.

## Conclusions

Despite these limitations, there are several areas where we are extending our research. First, the screening criteria used in the big data system were formulated based on consultations with public health experts and clinical practitioners. Moving forward, we plan to incorporate the implementation of artificial intelligence algorithms, such as deep learning, to refine the screening criteria to improve the detection rate. Second, even though our big data system was able to identify potential dengue cases, this information was not made available during face-to-face consultations. To improve the speed of detection, we aim to make the data available to doctors so that they can correctly diagnose patients. Third, we are expanding our big data system to include other data sources. In the medical area, we aim to include other forms of administrative health data such as insurance data and pharmacy transaction data [4]. We are also working on making our big data system interoperable with healthcare systems outside of Yinzhou. In that way, even if a person travels out of Yinzhou to receive treatment, his medical records would be available to the medical facility he visited to aid healthcare providers in making the right medical diagnosis. Besides health data, we are seeking to integrate non-medical data sources, such as individuals’ travel data as well as their mobile payments, to improve the efficacy of contact-tracing and epidemic surveillance. Finally, the screening criteria was developed in consultation with a panel of public health and medical experts so as to be sensitive to symptoms of infectious diseases in Yinzhou. Future research should incorporate both international and local contexts when developing screening criteria.

We have demonstrated through our three case studies that an integrated health big data approach is essential and effective for infectious disease control and management, and can potentially result in more lives saved and cost savings for the government and other decision makers. The ability of take advantage of health big data to improve infectious disease surveillance relies on a multi-party coordination and a concerted effort, as well as and the ability of government agencies, health organizations, hospitals and clinics, and private sectors to pull health data together and analyze them in a timely manner [10], provided that issues of data interoperability and privacy are adequately addressed [28].

## Data Availability

The datasets generated during and/or analyzed during the current study are not publicly available but are available from the corresponding author upon reasonable request.

## Abbreviations

IHBDP: Integrated health big data platform
EHRs: Electronic health records
CDC: Centers for Disease Control and Prevention
GDP: Gross Domestic Product
TB: Pulmonary tuberculosis
IDC: Internet Database Connector
